# Competition within Introns: Splicing Wins over Polyadenylation via a General Mechanism

**Published:** 2013

**Authors:** M. Tikhonov, P. Georgiev, O. Maksimenko

**Affiliations:** Department of the Control of Genetic Processes, Institute of Gene Biology, Russian Academy of Sciences, 34/5 Vavilova Str., Moscow, Russia, 119334

**Keywords:** transcription termination, splicing, polyadenylation signals, exon, intron

## Abstract

Most eukaryotic messenger RNAs are capped, spliced, and polyadenylated via co-transcriptional processes that are coupled to each other and to the transcription machinery. Coordination of these processes ensures correct RNA maturation and provides for the diversity of the transcribed isoforms. Thus, RNA processing is a chain of events in which the completion of one event is coupled to the initiation of the next one. In this context, the relationship between splicing and polyadenylation is an important aspect of gene regulation. We have found that cryptic polyadenylation signals are widely distributed over the intron sequences of *Drosophila melanogaster*. As shown by analyzing the distribution of genes arranged in a nested pattern, where one gene is fully located within an intron of another gene, overlapping of putative polyadenylation signals is a fairly common event affecting about 17% of all genes. Here we show that polyadenylation signals are silenced within introns: the poly(A) signal is utilized in the exonic but not in the intronic regions of the transcript. The transcription does not end within the introns, either in a transient reporter system or in the genomic context, while deletion of the 5'-splice site restores their functionality. According to a full *Drosophila *transcriptome analysis, utilization of intronic polyadenylation signals occurs very rarely and such events are likely to be inducible. These results confirm that the transcription apparatus ignores premature polyadenylation signals for as long as they are intronic.

## INTRODUCTION

During maturation, eukaryotic messenger RN As undergo capping, splicing, and polyadenylation, and the molecular machineries responsible for these modifications are coupled both to each other and to the transcriptional apparatus [1-5]. Collaboration between these machineries is confirmed by the existence of splicing proteins and the cleavage and polyadenylation proteins that can be recruited to RN A polymerase II (RN AP II) *in vitro *prior to transcription and then transferred to the RN A [6, 7]. The process of terminal exon definition is another example of such collaboration. It has been shown that recognition of the 3'-splice site (3'SS) of the gene’s last intron strongly improves the efficiency of the downstream polyadenylation site [8-10] and that the 3'-SS-associated factor U2AF enhances the function of the polyadenylation site by direct molecular contacts with poly(A) polymerase [11]. Similarly, the protein components of U2snRN P that associate with the 3'SS and nearby lariat branch point help to enhance downstream 3'-end processing through interactions with CPSF (cleavage and polyadenylation specificity factor) [12]. U2AF65 (splicing factor U2AF 65 kDa subunit) stimulates pre-mRN A 3'-end processing via the interaction of its arginine/serine-rich region with an RS-like alternating charge domain of the 59 kDa subunit of the human cleavage factor I (CF Im) [13]. It is likely that poly(A) site cleavage is followed by polyadenylation of the 3'-end and, finally, by splicing of the last intron [14], and that poly(A) addition triggers RN A release from the polymerase only after being licensed by splicing [15]. Another relevant fact is that U1 snRN P, an essential component in defining the 5'-splice site, interacts with mammalian polyadenylation cleavage factor I (CF Im) [16]. These data provide evidence for the interrelation of all processes involved in gene transcription, including its initiation, transcript elongation, splicing, and polyadenylation.

Polyadenylation signals (PASs) are not complex, and it appears that these elements are not confined to the 3'-UTR s of genes but occur throughout the genome, including the 5'-UTR s [17]. Premature PAS utilization may result in gene dysfunction and, therefore, must be prevented. It has been discovered that PASs become functionally silent when they are positioned close to transcription start sites in either *Drosophila *or human cells [17]. PAS were also found upstream of 5’ splice sites, and point-mutated splice donor activates an upstream cryptic polyadenylation (CpA) site [18]. In the bovine papilloma virus, utilization of the late PAS at earlier stages of infection is prevented by the presence of a closely positioned, upstream 5'SS. Recognition of this 5'SS by U1snRN P blocks poly(A) polymerase activity at the late PAS by direct interaction with the 70K protein component of U1snRN P [19]. A recent whole genome study on the effect of functional U1 snRN P knockdown in HeLa cells has revealed cases of premature cleavage and polyadenylation in numerous premRN As at cryptic PASs, frequently in introns near the transcription start site [20]. Based on the fact of polyadenylation silencing by U1 snRN P it was suggested that recruiting of U1 snRN P to the target pre-mRN A inhibits poly(A)-tail addition, causing degradation of such RN A species in the nucleus [21, 22]. Quantitative analysis of a number of mRN A variants generated by intronic PASs suggests that the intronic polyadenylation activity can vary under different cellular conditions [23]. For example, the level of U1 snRN P defines the length of the transcript and the ability to utilize premature PAS within introns and in distal 3’UTR s [24]. In view of the abovementioned data, it is likely that splicing and polyadenylation within introns interact in a competitive manner.

In this study, we focus on the relationships between splicing and polyadenylation in cases of intronic location of PASs in the *Drosophila *genome. Our results confirm the wide distributions of these signals within the introns of genes and show that PASs are silenced within introns in a transient reporter system, as well as in the genomic context. Meanwhile, deletion of 5'SS restores polyadenylation activity. Analysis of RN A-seq data for different cell lines and development stages of* Drosophila *provides evidence of a switch between synthesized isoforms in case of alternative 3'-exon-inclusion transcripts.

## EXPERIMENTAL

**Bioinformatic poly(A) signal prediction**

The PolyA_SVM program was used to identify putative polyadenylation sites [25]. This program was previously shown to be suitable for site prediction in *Drosophila* [17]. The program searches for poly(A) signals by using a window-based scoring scheme to evaluate the fitness of 15 *cis*-elements identified from known human poly(A) signals [25]. The whole data set of *D. melanogaster *annotated introns is available from FlyBase [26]. The probability for an element to be a poly(A) signal is characterized by the E-value (the lower the value, the higher the probability). The output was programmatically sorted into three categories: “site is present,” “site is not found,” and “input sequence is too short.”

**Search for nested genes**

*Drosophila melanogaster *genome annotation data from FlyBase [26] were used to parse the coordinates of genes and introns. A gene was considered to be nested if its start coordinate was greater than the start coordinate of the corresponding intron and its end coordinate was smaller than the end coordinate of the intron.

**Construction of plasmid reporter system**

The bicistronic plasmid constructs were generated in pAc5.1/V5-His B (Invitrogen). The firefly and *Renilla* luciferase sequences were taken from the pGL3Basic and pRL-CMV vectors (Promega), respectively. The* reaper *gene IRE S was amplified from genomic DNA and cloned upstream of the firefly luciferase sequence. The SV40 terminator sequence was taken from the pAc5.1/V5-His B vector. The intron and terminator of the *yellow *gene were taken from a 8-kb gene fragment kindly provided by P. Geyer. The polyadenylation signals of the *nop5 *and *eIF6 *genes were amplified from genomic DNA. To produce the artificial intron (AI), oligonucleotides containing the desired sites were synthesized. The *lac*Z CDS region was taken as a linker sequence between the donor and acceptor splicing sites.

**Cell culture, transfection, RNA purification, and dual luciferase assay**

*Drosophila *S2 cells were grown in a SFX medium (Hy- Clone) at 25°C. Transfection of plasmids was performed with the Cellfectin II reagent (Invitrogen) according to the manufacturer’s instructions. Typically, cells were transfected in six-well plates and grown for 24 to 48 h before harvesting.

Total RN A was extracted from the transfected cells using the TR I reagent (Ambion) according to the manufacturer’s instructions. To fractionate nuclear and cytoplasmic RN As, S2 cells collected from a 100 mm dish were washed with PBS, pelleted, and re-suspended in 100 μl of TD (0.8% NaCl, 0.028M KCl, 0.01% Na2HPO4, 0.3% Tris-HCl; pH 7.4-7.5). The mixture was supplemented with 100 μl of TD with 1% NP-40 and SUPER - ase-In (Ambion) and kept on ice for 5 min. The sample was then centrifuged, and the supernatant fluid was used to isolate the cytoplasmic RN A fraction with the TR I reagent (Ambion). The nuclear pellet was re-suspended in 200 μl of TD with 0.5% NP-40 and SUPER - ase-In, incubated on ice, and centrifuged again. The nuclear RN A fraction was isolated from the pellet using the TR I reagent.

The dual luciferase assay was performed with the Firefly & Renilla Luciferase Assay Kit (Biotium).

**RNA analysis**

For Northern analysis, 5-20 μg of total RN A was separated in 1% agarose gel in the presence of formaldehyde and blotted onto a positively charged nylon membrane (BrightStar-Plus, Ambion) in a Trans-Blot SD Semi- Dry Electrophoretic Transfer Cell (Bio-Rad), which was followed by cross-linking under UV light. The regions of interest were amplified and cloned under the T7 promoter. The membranes were hybridized with *in vitro *synthesized RN A probes (Ambion MEGAshortscript and MAXIscript kits) with biotin-16-UT P (Roche) inclusion and examined using a Chemiluminescent Nucleic Acid Detection Module (Thermo Scientific).

Real-time PCR experiments were performed with reverse transcription products. RN A was treated with two units of Turbo DNase I (Ambion) for 30 min at 37°C to eliminate genomic DNA. The synthesis of cDNA was performed using ArrayScript reverse transcriptase (Ambion) in a reaction mixture containing 5 μg of RN A and random hexamer primers. Specific cDNA fragments were quantitatively analyzed by real-time PCR using a CFX96 Thermal Cycler (Bio-Rad). At least three independent experiments with each primer set were performed for three independent RN A samples. Relative levels of mRN A expression were calculated using the cycle threshold method.

**Analysis of isoform expression pattern based on RNA-seq data**

All procedures were performed programmatically using Java language. We used the genome annotation data from FlyBase (release 5.40) to search for transcripts ending within an intron. These were transcripts with the last exon starting upstream of the donor splice site and the end of the transcript located between the splicing signals. The genes overlapping with each other were then excluded from consideration, and analysis was confined to the genes with transcripts of only two forms, the first being spliced and the second ending within an intron. SAM files with RN A-seq information were obtained from modENC ODE for 30 development stages and 4 cell lines, and their reads were superimposed and matched up with the structural features of genes. The mean value of the read density for the 3’-exon was taken to reflect the level of the spliced form. The level of the intron-cleaved/polyadenylated isoform was calculated as the difference between the mean values of the read density for the region between the 5' splice site and the transcript end and for the region between the transcript end and the 3' splice site (probably corresponding to unspliced RN A). A heat map was created to visualize the expression pattern of each isoform and the ratio of the intron-intron-cleaved/ polyadenilated isoform to the sum of two forms.

## RESULTS

**PASs are widely present in** *Drosophila* **introns**

As noted above, PASs appear to be widely distributed throughout the genome, and their premature utilization may result in gene dysfunction. Hence, there should be mechanisms for preventing utilization of inappropriate PASs. Previously, polyadenylation silencing was shown to take place in the 5'UTR s of genes [17]. The occurrence of any inappropriate signals in coding sequences is prevented by selection pressure, but noncoding intronic sequences are prone to change and can contain premature PASs. We checked how widely such signals are distributed within the intronic sequences of* Drosophila *genes. For this purpose, we used the PolyA_ SVM program designed for the analysis and prediction of mRN A polyadenylation sites by a Support Vector Machine [25]. This program was previously shown to be suitable for site prediction in *Drosophila *[17].

**Fig. 1:**
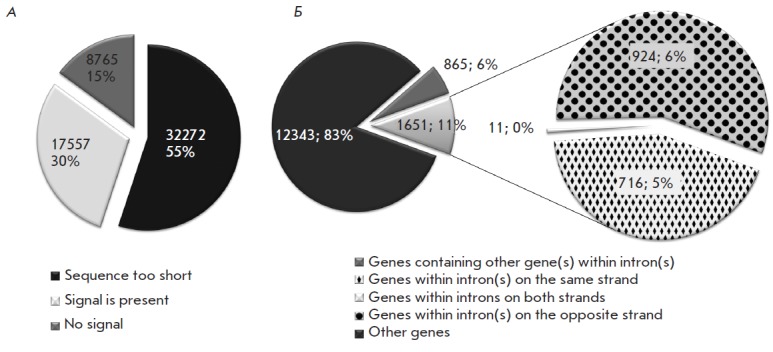
Poly(A) signals are widely distributed in *Drosophila *introns. A. Pie chart showing the proportion of introns with or without poly(A) signals and of short introns as predicted by the PolyA_SVM program. B. Pie charts showing the proportions of the genes containing other gene(s) within introns (host genes) and of genes enclosed into introns (nested genes) divided into three groups according to their location on DNA strands

The full set of 58 594 *Drosophila *intron sequences was taken from the FlyBase genome annotation database, release 5.34 [26]. Approximately 55% of these sequences were shorter than 120 bp, the minimum length necessary for running the above program (Figure 1A); two-thirds of the other sequences (about 30% of the total set) were predicted to contain one or more PAS copies. Thus, putative PASs were found to be widely distributed in *Drosophila *introns. There are two functional possibilities for such intronic PASs: they can be either silenced or utilized. In the first case, the signal has no effect on transcription and subsequent splicing; in the second case, transcription is untimely interrupted by the signal.

In this context, it was of interest to study the genes arranged in a nested pattern, where one (‘nested’) gene is located within an intron of another (‘host’) gene. In this case, a PAS from the nested gene should have no effect on the host gene transcription. The main difference (except in length) between the transcripts of the host and nested genes at the time of read-through of the PAS of the latter is that the host gene transcript contains the 5' splice site. We analyzed the distribution of such genes using the FlyBase data [26] (*Fig. 1* B). The coordinates of genes and introns were used for searching for cases where one gene is fully located within an intron of another gene. We determined 865 host genes that contained 1,651 nested genes within the introns on both strands. Among them, 727 nested genes had the same transcription direction as the host genes, and, therefore, their putative regulatory elements overlapped with those of the latter. Thus, we found that PASs are widely distributed over the intron sequences and that overlapping of putative PASs signals is a fairly common event affecting about 17% of genes.

**Experimental evidence for PAS utilization in exonic, but not intronic, transcript regions**

**Fig. 2:**
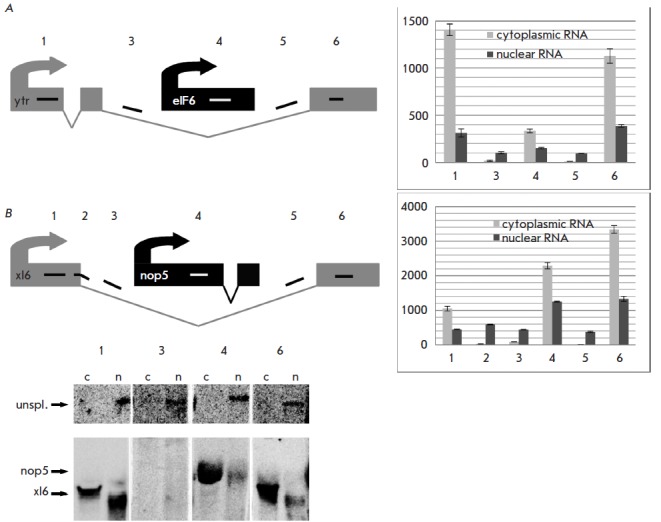
Poly(A) signals are utilized in exonic, but not intronic transcript regions. In schemes of experiments with (A) *ytr– eIF6 *and (B) *xl6–nop5 *gene pairs, exons and introns are shown as boxes and angles (V), respectively; short numbered lines indicate the regions recognized by probes. Histograms show the levels of nuclear and cytoplasmic RNAs in these regions. Error bars represent s.d. (n=3). Northern-blot analysis of nuclear (n) and cytoplasmic (c) RNAs isolated from S2 cells and hybridized with probes to the *xl6–nop5 *gene pair is shown at the bottom

As shown above, the transcription machinery frequently stumbles due to untimely PASs within introns. If these intronic PASs are indeed utilized, this could be shown on the pairs of nested and host genes. To test whether the PAS from the nested gene influences the transcription of the host gene, we performed a RT -PCR analysis for two gene pairs, *ytr*-*eIF6 *and *xl6*-*nop5* (*Fig. 2*). The main criterion for selecting these genes was the high expression level of both nested and host genes in the *Drosophila *S2 cell line. The expression profiles were obtained from the modENC ODE RN A-seq database [27, 28]. The analysis was performed with nuclear and cytoplasmic RN A samples using probes for sequences located in the gene regions shown in *Fig. 2*. While the cytoplasmic fraction contained fully processed mRN A, the nuclear fraction additionally included intermediates and decay products, and its analysis allowed us to detect transcripts that had not been processed to their mature form and released into the cytoplasm.

In cytoplasmic samples, the RT -PCR analysis revealed high RN A levels only for the exonic regions of the test genes (*Fig. 2*; probes 1, 4, 6), while intronic regions were expressed at the background level (*Fig. 2*; probes 3, 5). In nuclear samples, however, RN A levels for the intronic are detectable, indicating the presence of long, unspliced *ytr* (Fig. 2A) or *xl6 *transcripts (Fig. 2B). The absence of significant variations between intronic regions (points 3 and 5) and the last exon (point 6) is evidence that no premature cleavage/polyadenylation of *ytr *or *xl6 *occurred within the intron and that the respective *eIF6 *or *nop5 *transcripts were processed at their own PASs. Notably it recognizes only its own polyadenylation signal for each transcript.

We simultaneously performed a Northern blot analysis for the *xl6*-*nop5 *pair to reveal bands recognized by all exonic and one intronic probes within the* xl6*/*nop5 *gene span (Fig. 2B). In the nuclear fraction, all probes detected the presence of long RN A (at the detection limit), which corresponded to an unspliced readthrough* xl6 *gene product. In both nuclear and cytoplasmic fractions, we also observed signals from exonic probes, which corresponded to the processed forms of* xl6 *(probes 1 and 6) and *nop5 *(probe 4). It should be noted that the Northern blot analysis did not reveal the form of *xl6 *transcript cleaved/polyadenylated at the *nop5 *PAS, which is located in the intronic region of the nascent *xl6 *transcript. The *xl6 *transcript was only cleaved/polyadenylated at its own PAS located in the corresponding exonic sequence.

Summarizing the results of Northern blotting and RT -PCR allows us to conclude that the transcription machinery of the host gene ignores the intronic PAS, whereas that of the nested gene successfully utilizes the same signal, which in this case is exonic.

**PAS inserted in an intron is functionally disabled**

**Fig. 3:**
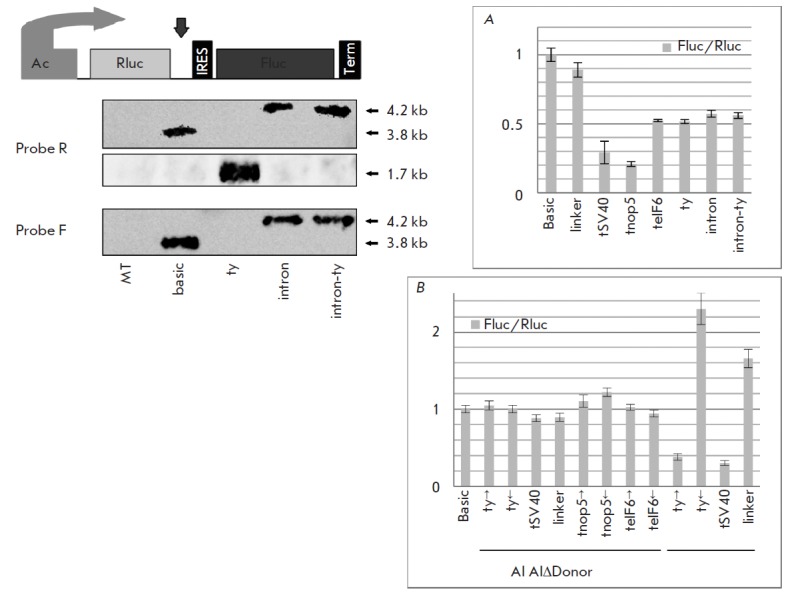
Plasmid reporter system confirms that poly(A) signal inserted in an intron is functionally disabled. This bicistronic reporter system is based on *Renilla *luciferase (Rluc) and *Firefly *luciferase (Fluc) coding sequences driven by the single* Drosophila actin *5C promoter, with the IRES sequence inserted between the luciferase sequences. The arrow shows the site of insertion of the late SV40 PAS (tSV40) and PASs from genes *nop5 *(tnop5), *eIF6 *(teIF6), and *yellow *(ty). The intron from the *yellow *gene and this intron with a ty insertion were cloned at the same position. The Fluc/Rluc ratios for these constructs are shown in histogram **A**. Error bars represent s.d. (n=5). Northern blot analysis of total RNA from mock-treated (MT) and transfected S2 cells with probes to both luciferases confirms the results of luciferase assay. Histogram **B **shows the Fluc/Rluc ratios for artificial intron (AI)-based constructs containing the complete AI and AI with donor site deletion (AI ΔDonor). Error bars represent s.d. (n=3)

Analysis of endogenous cleavage/polyadenylation events characterizes gene functioning “as is” and does not provide enough freedom for making alterations in this process. Therefore, we turned to the plasmid reporter system to analyze PAS functioning in the *Drosophila* S2 cell line. This bicistronic system was based on the *Renilla *luciferase (Rluc) and firefly luciferase (Fluc) coding sequences driven by the single *Drosophila actin 5C *promoter, with the IRE S sequence from the* Drosophila reaper *gene [29] being inserted between the luciferase sequences (Fig. 3).

We expected that if the test PAS was functional, a monocistronic Rluc mRN A would be produced; if this PAS was nonfunctional or weakly functional, a longer mRN A would be generated, extending to the SV40 PAS located downstream of the Fluc sequence. The plasmid constructs were transfected into *Drosophila *S2 cells and analyzed 24 to 48 h after transfection by means of dual luciferase assay. The amount of long bicistronic mRN A relative to the total mRN A from the construct was estimated from the Fluc/Rluc ratio.

In the first set of constructs, which was used to measure the basal cleavage/polyadenylation activity, the late PAS from the SV40 virus (tSV40) and PASs from the *nop5 *(tnop5), *eIF6 *(teIF6), *yellow *(ty) genes were inserted downstream of the first cistron. The basic construct without insertion and the construct with the linker sequence (without PAS) of the same length as the PASs were used as controls. All the above-mentioned PASs proved to reduce the Fluc/Rluc ratio, indicating that the transcripts were cleaved/polyadenylated after the first luciferase (Rluc) (Fig. 3 A). For basic and ty constructs, we performed a Northern blot analysis of isolated total RN A with probes R and F recognizing the Rluc and Fluc sequences, respectively. The results confirmed that the ty construct generated shorter transcripts: as detected with probe R, 1.7 kb vs. 3.8 kb in the basic construct (Fig. 3).

The second set of constructs was aimed at measuring the cleavage/polyadenylation activity in the intronic sequence from the *Drosophila yellow *gene. It included plasmids with this intron and with the ty PAS inserted in the intron. The dual luciferase assay showed that intron insertion reduced the Fluc/Rluc ratio, compared to the basic construct (Fig. 3 A). We attributed this observation to the change in the efficiency of IRE Sdependent translation initiation. Meanwhile, the ty insertion in the *yellow *intron did not change the ratio characteristic of the intron construct without this insertion. The Northern blot analysis for these constructs was performed with probes to both luciferases (Fig. 3). Probe F generated signals for long bicistronic transcripts. Signals were obtained for basic and two introncontaining constructs, with the transcript length of intron- containing constructs corresponding to a spliced variant (4.2 kb). Probe R generated a signal for all RN A transcribed from the construct promoter. For the ty construct, a short form of monocistronic transcript was only detected (1.7 kb). For basic and intron-containing constructs, the bands obtained with probe R were the same as those detected with probe F. Remarkably, there were no short forms of transcripts from the construct with the ty insertion within the intron.

Thus, we showed that the transcript was processed in all constructs where a PAS was placed in the exonic sequence. PAS insertion in the *yellow *intron did not interrupt transcription, and only a spliced form of RN A was detected.

The intron from the *yellow *gene, a long sequence, could contain unknown putative regulatory elements having an effect on transcription. Therefore, we constructed an artificial intron (AI) that contained no other regulatory elements except the minimum set of splicing signals: the donor site, acceptor site, branch point, and poly(T/C) region. A fragment of the *lac*Z CDS was used as a linker between the splicing signals. Based on the basic bicistronic reporter, we designed constructs containing different PASs or the linker sequence within the AI. Four PASs were tested in this way: ty, tSV40, tnop5, and teIF6 (Fig. 3 B). As negative controls, ty, tnop5, and teIF6 were cloned at the same position in reverse orientation. Since SV40 poly(A) is functional in both orientations, a *lac*Z CDS fragment of equal length was taken as a negative control in this case. The Fluc/Rluc ratio in constructs containing polyadenylation signals in the direct orientation was not changed significantly compared to that in the control constructs with these signals in the reverse orientation or with the* lac*Z linker (Fig. 3 B). Thus, no events of intronic-PAS utilization were observed in this case, as well as in the experiments with the endogenous gene pairs and the model system based on the *yellow *gene intron.

To confirm cleavage/polyadenylation silencing within the intronic sequence, we performed a similar experiment with AI variants of ty and tSV40 constructs in which the donor splice site was deleted. Expectedly, if this deletion turned the AI into exon extension, then PAS would be utilized. As shown in Fig. 3 B, the Fluc/Rluc ratios for PAS-containing plasmids were lower. Therefore, in the absence of the donor splice site, a short monocistronic transcript isoform is generated due to the functioning of the first PAS. A reverse experiment, where mutation creates a functional 5’ splice site and that its recognition by the spliceosomal component U1 snRN P causes suppression of 3' end formation, was also described [30, 31].

**PAS utilization within intron is a rare event in the genome and appears to be inducible**

**Fig. 4:**
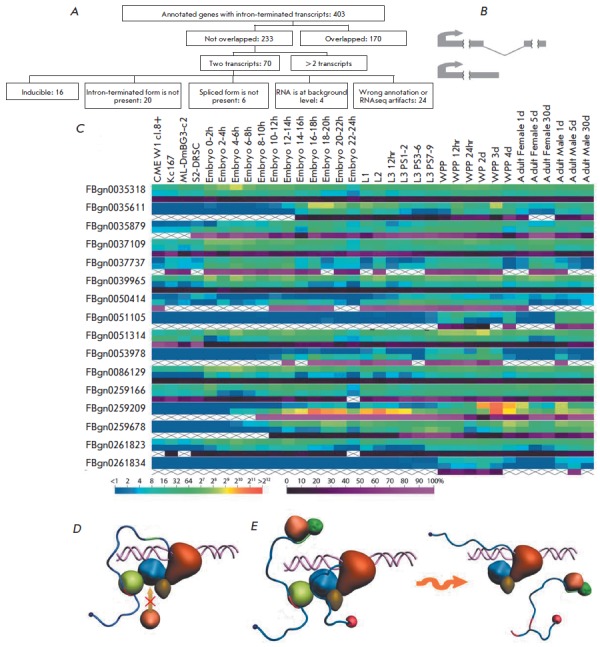
Cleavage/polyadenylation within an intron is a rare event in the genome and appears to be inducible. A. Scheme of searching for genes with intron-cleaved/polyadenylated transcript isoforms. B. Scheme of selected genes transcripts, with one isoform cleaved/polyadenylated within the intron of the other isoform. C. Heat map illustrating the expression patterns of the two transcript isoforms from 15 genes in 4 cell lines and at 30 development stages. For each gene, the top line refers to the spliced form; the middle line, to the intron-terminated isoform; and the bottom line, to the ratio between the intron-terminated isoform and the total gene mRNA. Color scales at the bottom characterize the level of isoform expression in RNA-seq read density units (on the left) and the proportion of the intron-terminated isoform (on the right). D-E. Two potential models for intronic PAS bypassing. D. In the “antitermination” model, PAS is inaccessible to cleavage/polyadenylation proteins due to competitive binding of splicing and termination components to the elongation complex, in which splicing wins over polyadenylation because of its earlier functional readiness during transcription. E. In the “kinetic model,” PAS is accessible, but moving polymerase reaches the 3' splice site quickly enough to initiate the splicing reaction; an introduced break remains in the cut-out intermediate and does not allow exonuclease-based transcription termination

We found that polyadenylation occurs within introns neither in the genome nor in transgenic constructs. It is known, however, that in case of alternative 3'-exon inclusion the transcript should be interrupted within an intron (Fig. 4 A, B). Using the FlyBase genome annotation data [26], we checked how often the annotated transcripts ended within the introns of another transcript isoform and found 403 genes organized in this way. To analyze the expression pattern of isoforms, we used RN A-seq data on different cell lines and developmental stages that are available from the modENC ODE project [27, 28]. We excluded 170 genes whose transcripts overlapped with each other at their boundaries, because in this case it was impossible to determine the gene from which a given sequence was transcribed. Then we chose 70 genes with transcripts of only two forms, the first being spliced and the second ending within an intron (Fig. 4A). The proportion of the intron-cleaved/polyadenylated form was estimated as the ratio between its level and the sum of the two RN A forms.

According to the calculated levels and ratios, we sorted the chosen genes into several groups. The first major group consisted of 20 genes in which transcripts utilizing PAS within introns were not detected or their level was close to the baseline noise. It may well be that such genes do not produce intron-cleaved/polyadenylated transcripts or produce a very small amount of them. The second group included six genes showing no detectable splicing events and impugning the existence of 3'-exons. The third group consisted of 16 genes with transcriptional switching between the isoforms, which is likely to be inducible (Fig. 4C). The level of each isoform and the ratio between them change during development or in different cell lines. In addition, we sorted out a group of genes with apparently erroneous annotation. Thus, our observations show that PAS utilization within introns is a very rare occurance. According to the genome annotation data, only 403 genes possibly have transcripts ending within introns. In fact, only 20 out of the 70 genes included in the analysis produced such transcripts at a near-baseline level and only 16 genes produced both transcript isoforms, with their levels and ratio changing during development or in different cell lines.

## DISCUSSION

Our premise in this study was that inappropriate PASs in the intronic sequences of genes are prevented from utilization. This phenomenon was described earlier as finding functional cryptic PASs in introns after U1 sn- RN P knockdown in HeLa cells [20]. To begin with, we checked the occurrence frequency of such signals in* Drosophila *and found cryptic PASs to be widely distributed over the introns (about 30% of all introns). We then turned our attention to the cases where one gene is located within an intron of another gene and, therefore, the transcription machinery of the latter needs to read through premature PASs from the gene nested in its intron. Our experiments showed that such gene architecture does not result in the functional overlap of PASs and that the transcription machinery of the host gene takes no notice of intronic PASs from the nested gene. Furthermore, we did not observe transcript generated at intronic polyadenylation sites in experiments with the plasmid reporter system containing either an endogenous intron from the *yellow *gene or an artificially constructed intron. It is noteworthy that deletion of the donor splice site in this reporter system proved to restore the functionality of PAS. Finally, the full transcriptome analysis showed that transcripts of the isoform resulting from intronic PAS utilization are rarely expressed in *Drosophila *and that the ratio between these isoforms and the spliced ones varies during development.

Summarizing our findings and the previously obtained [19, 20] data, we can draw a conclusion that transcription is generally not interrupted at intronic PASs. Exceptions to this rule are rare, which confirms its validity and indicates that there should be some additional conditions for the activation of PASs within introns. It is noteworthy that, among genes with alternative 3'-exon inclusion transcripts, we found only 16 genes producing two transcript isoforms at the same time, one spliced and the other ending within an intron. In our opinion, inducible switching between the two isoforms takes place in this case.

There are two models that can potentially explain this phenomenon. The first one, the “antitermination” model, is based on the recent data on coupling between the splicing machinery and the cleavage/polyadenylation complex [14, 19, 20]. It is possible that splicing and polyadenylation events interact in a competitive manner, with the former prevailing over the latter (Fig. 4D). The effect may be mediated by direct protein-protein interactions: for example, by competitive binding of splicing and cleavage/polyadenylation components to the CT D of RN AP II or inactivation of cleavage/polyadenylation components by splicing factors. For example, snRN P inhibits PAP through a direct interaction between U1 70K and PAP [19]. After recognition of the donor splice site by U1snRN P, the elongation complex of RN AP II becomes inaccessible to the C/P components. Splicing wins over polyadenylation because the components of its machinery are assembled into a functional complex at earlier stages of the transcription process. Meanwhile, utilization of PAS within introns may be induced in some cases by the general mechanisms involved in the regulation of alternative splicing (such as masking of the donor splicing site by a regulatory protein or complementary RN A binding, changes in chromatin status) or by the level of U1 snRN P as described in [24].

The second one, the “kinetic model,” is based on the assumption that RN AP II continues to move after stumbling on a PAS [32, 33]; as a result, it successfully arrives to the acceptor splice site, initiating the splicing reaction with lariat formation (Fig. 4E). In this variant, cleavage and polyadenylation reactions take place but do not affect mRN A maturation, since the lariat intermediate is cut out. As shown in [34], the exons flanking the intron that has been engineered to be a co-transcriptional self-cleavage site (CoTC ) are accurately and efficiently spliced together. So we may assume that PASs within introns act in a similar way to CoTC . In this model, the “choice” of the transcript isoform can be regulated not only by masking the donor splice site but also by changes in the rate of RN AP II movement depending on CpG methylation and chromatin status. In the general case, RN AP II moving at a high rate manages to reach the acceptor splice site before being displaced by 5'-3'-exonuclease, which is recruited onto nascent RN A after cleavage events at the intronic PAS. If RN AP II is paused or slowed down, then transcription is terminated, which results in short isoform production.

Another possibility of the products obtained utilizing intronic PAS is degradation. However, no products generated on PAS within introns were detected in our study. Hence, if these events occur, either degradation is very quick or the amount of produced RN A is extremely low.

The observed phenomenon contributes to the understanding of the transcription logic, indicating that transcript 3’-end formation takes place only at appropriate positions. The molecular mechanisms of PAS skipping or, on the contrary, rare activation require special study.
